# Nonmagnetic single-molecule spin-filter based on quantum interference

**DOI:** 10.1038/s41467-019-13537-z

**Published:** 2019-12-05

**Authors:** Atindra Nath Pal, Dongzhe Li, Soumyajit Sarkar, Sudipto Chakrabarti, Ayelet Vilan, Leeor Kronik, Alexander Smogunov, Oren Tal

**Affiliations:** 1 Department of Chemical and Biological Physics, Weizmann Institute of Science, Rehovot, Israel; 2 Department of Condensed Matter Physics and Material Sciences, S. N. Bose National Centre for Basic Sciences, Block JD, Sector III, Salt Lake, Kolkata, India; 3 Department of Physics, University of Konstanz, Konstanz, Germany; 4 Department of Materials and Interfaces, Weizmann Institute of Science, Rehovot, Israel; 5 SPEC, CEA, CNRS, Université Paris-Saclay, CEA Saclay, Cedex Gif-sur-Yvette, France

**Keywords:** Quantum chemistry, Electronic properties and materials, Molecular electronics, Organic-inorganic nanostructures, Spintronics

## Abstract

Key spin transport phenomena, including magnetoresistance and spin transfer torque, cannot be activated without spin-polarized currents, in which one electron spin is dominant. At the nanoscale, the relevant length-scale for modern spintronics, spin current generation is rather limited due to unwanted contributions from poorly spin-polarized frontier states in ferromagnetic electrodes, or too short length-scales for efficient spin splitting by spin-orbit interaction and magnetic fields. Here, we show that spin-polarized currents can be generated in silver-vanadocene-silver single molecule junctions without magnetic components or magnetic fields. In some cases, the measured spin currents approach the limit of ideal ballistic spin transport. Comparison between conductance and shot-noise measurements to detailed calculations reveals a mechanism based on spin-dependent quantum interference that yields very efficient spin filtering. Our findings pave the way for nanoscale spintronics based on quantum interference, with the advantages of low sensitivity to decoherence effects and the freedom to use non-magnetic materials.

## Abstract (web summary)

Quantum interference can be used to control electronic transport with high sensitivity at the nanoscale. Pal et al. show that without the need for magnetic materials, quantum interference can also filter spin transport approaching the limit of ideal spin-polarized ballistic transport in molecular junctions.

## Introduction

Quantum interference is a fundamental quantum mechanical phenomenon with a variety of implications for metrology^1^, quantum information processing^2^, and chemical processes^3,4^. In nanoscale electronic devices, electron quantum interference is an attractive effect because it can be used to control electronic transport using strategies that are not accessible by traditional electronics. In recent years, quantum interference has been studied intensively in molecular junctions based on molecules suspended between two electrodes^5–19^. In a series of experiments complemented by calculations, it was shown that destructive interference between different current pathways across the molecular bridge efficiently suppresses the overall charge transport in the studied molecular junction^10–18^. Interestingly, the structure of the molecular bridge can be optimized to promote efficient destructive interference at the relevant energy for electronic transport, such that the bridging molecule can be a better insulator than a vacuum gap^16^. In contrast, constructive interference can cause the measured total conductance of two transport pathways across a molecular junction to be more than twice the conductance of each pathway^19^. Despite extensive experimental study of quantum interference in molecular junctions, the influence of quantum interference on spin transport in such junctions has not been demonstrated.

Here, we show that highly spin-polarized currents can be generated in paramagnetic molecular junctions without magnetic components or magnetic fields. Moreover, in some cases the obtained current approaches the limit of ideal ballistic spin transport. Specifically, using conductance and shot-noise measurements we detect spin-polarized currents in molecular junctions based on silver electrodes bridged by a vanadocene molecule. Some of the junctions show spin transport close to one quantum of spin conductance, which is dominated by an almost fully-open spin-polarized transmission channel. Detailed comparison between experimental findings and transport calculations reveals a mechanism based on spin-dependent quantum interference that yields very efficient spin filtering. Our findings pave the way for nanoscale spintronics based on quantum interference, with the advantages of low sensitivity to decoherence effects at the nanometer scale, and the freedom to use non-magnetic materials for faster, power-saving spin manipulations.

## Results

### Conductance measurements

**Fig. 1:**
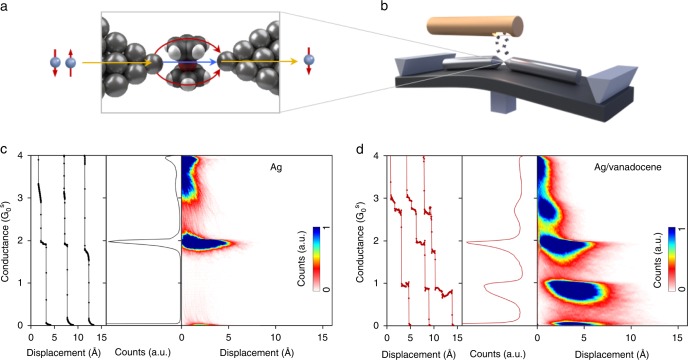
Measured conductance of bare silver atomic-scale junctions and Ag/vanadocene molecular junctions. **a** Schematic representation of spin-filtering by interfering current pathways in a Ag/vanadocene single-molecule junction. **b** Schematics of the break-junction setup. **c** Characterization of bare silver atomic junctions. Left panel: examples for conductance versus inter-electrode displacement traces recorded at a bias voltage of 100 mV, with traces shifted in the displacement axis for clarity; Middle panel: conductance histogram constructed from 5,000 conductance traces; Right panel: conductance–displacement density plot based on the same conductance traces. **d** Similar characterization for Ag/vanadocene molecular junctions, with histograms based on the same number of traces. Zero displacement is determined for each conductance trace by the first displacement point with a conductance value below 4 $G_0^{\mathrm{s}}$. Information on the measurement circuit can be found in Supplementary Note 1.

The first single-molecule junctions considered here are based on individual vanadocene molecules suspended between two silver electrodes (Fig. 1a). The vanadocene molecule has a sandwich structure, with a core vanadium ion located between two cyclopentadienyl conjugated rings. In the gas phase, vanadocene has a total spin of S = 3/2^20^, which is essentially preserved in the junction, as confirmed by our ab initio calculations.

We use a break-junction setup (Fig. 1b)^21^ to form in situ molecular junctions in cryogenic vacuum conditions^22^. Specifically, two clean silver electrodes are prepared by breaking a silver wire at 4.2 *K* into two segments with freshly exposed apices separated by a nanoscale gap (see Methods). Before the introduction of molecules, we verify the typical conductance of bare silver atomic junctions by repeatedly forming atomic-scale contacts between the electrode apices. Figure 1c, left panel shows examples for traces of conductance vs. inter electrodes displacement that were recorded during repeated breaking of the clean Ag junction. Before recording the next breaking conductance trace, the junction is structurally deformed by pushing the electrode apices against each other to promote sampling of different junction structures. During the breaking process the conductance decreases and eventually a single silver atom contact is formed as indicated by a typical conductance plateau at ~2 $G_0^{\mathrm{s}}$, ($G_0^{\mathrm{s}} = e^2/h$ is the spin-polarized conductance quantum, where *e* is the electron charge, and *h* is Planck’s constant)^21^. This value is given by two equal transmission channels for spin-up and down. Further elongation leads to junction breaking, followed by an abrupt conductance drop. The conductance histogram in Fig. 1c, middle panel, which is constructed from 5000 conductance traces, provides statistical information. The peak at ~2 $G_0^{\mathrm{s}}$ indicates that this is the most probable conductance of a silver atomic-scale junction, while the low conductance tail is the manifestation of tunneling transport after junction breaking. The conductance versus displacement density plot in Fig. 1c, right panel, presents the conductance evolution during the junction breaking process.

When vanadocene molecules are introduced to the junction from a local molecular source, additional plateaus appear at ~1 $G_0^{\mathrm{s}}$ (Fig. 1d, left panel). These plateaus indicate the formation of molecular junctions during the breaking process of the silver junction. Figure 1d, middle panel, presents a corresponding conductance histogram for the Ag/vanadocene junctions with a peak at ~2 $G_0^{\mathrm{s}}$, as in the case of bare silver atomic junctions, and a new peak at ~1 $G_0^{\mathrm{s}}$. The molecular origin of the lower conductance value is further verified by detecting the vibrational signature of the newly formed molecular junctions, using inelastic electron spectroscopy^23^. The conductance versus displacement density plot in Fig. 1d, right panel, shows the conductance evolution during the breaking process of the molecular junction. A conductance of 1 $G_0^{\mathrm{s}}$, has been often regarded (incorrectly) as the smoking gun of fully spin-polarized conductance, namely conductance of only one spin type. For quantum coherent conductors, the conductance *G* is given by the Landauer formalism^24^ as a sum of contributions from different spin-up and spin-down ($\sigma =\, \uparrow , \downarrow$) transmission channels, where the transmission of electrons via each spin-polarized channel has a probability $\tau _{i,\sigma }$ (*i* is the channel index), namely, $G = G_0^{\mathrm{s}}\mathop {\sum }\nolimits_{i,\sigma } \tau _{i,\sigma }$. The conductance is indeed 1 $G_0^{\mathrm{s}}$ for fully spin-polarized transport carried by a fully-open single spin channel ($\tau _\sigma = 1$). However, the same conductance can also be obtained by an arbitrary sum of partially open channels ($0\; <\; \tau _{{\mathrm{i}},{\upsigma}}\; <\; 1$), not necessarily of the same spin type. Thus, a conductance of 1 $G_0^{\mathrm{s}}$ is not a sufficient indicator for fully spin-polarized conductance and further information, such as the transmission probability of each spin-polarized channel, is required for assessing the degree of conduction spin-polarization (CSP).

### Shot-noise measurements

**Fig. 2:**
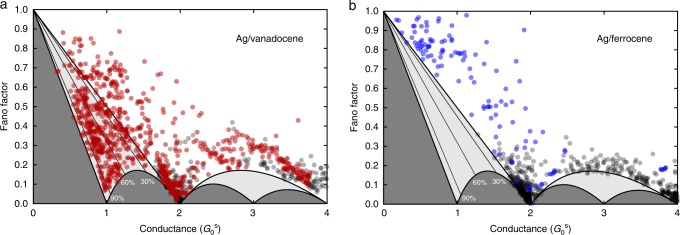
Fano factor for the studied molecular junctions and the bare silver atomic-scale junctions. **a** Fano factor (*F*) extracted by shot noise and conductance (*G*) measurements (see Supplementary Note 2) as a function of conductance for 900 different realizations of Ag/vanadocene molecular junctions (red), and similar measurements for 327 bare silver atomic-scale junctions (black). These data sets were taken independently of the data presented in Fig. 1. **b** The same for 123 Ag/ferrocene molecular junctions (blue), and 1,025 bare silver atomic-scale junctions (black). Data points that are located within the light-gray region imply spin-polarized conductance. The thick black curve separating the light-gray and dark gray areas provides the minimal Fano factor, where data accumulating on this line for a conductance of $G_0^{\mathrm{s}}$ or less indicate junctions with a single spin-polarized transmission channel and *P*_*G*_ = 100%. The thin black lines provide the minimal *P*_*G*_ for data below these lines. (*F,G*) data obtained for Ag/vanadocene junction clearly penetrate the light-gray region, indicating finite CSP, where for some of these data points *P*_*G*_ > 90%. In contrast, for Ag/ferrocene molecular junctions there is no penetration of data points into the light-gray region. The uncertainty due to systematic errors in our measurements is comparable to or slightly larger than the symbols’ diameter, as can be seen in Supplementary Fig. 3.

In molecular junctions^25,26^ and generally in a variety of spintronic devices^27–29^ the CSP, defined as $P_{\mathrm{G}} = \left| {(G_ \uparrow - G_ \downarrow )/(G_ \uparrow + G_ \downarrow )} \right|$, can be estimated under certain assumptions by magneto-transport measurements in a spin-valve configuration^29^. However, this approach is irrelevant for the studied molecular junction, because such measurements require magnetic electrodes. Instead, we turn to shot-noise measurements that provide useful information about the CSP and the transmission probabilities of spin-polarized channels^30–33^. Electronic shot-noise stems from the discreteness of electrons. When current is transmitted across a quantum conductor, each incident electron can be transmitted with a probability $\tau _{i,\sigma }$ or backscatter with a probability ($1 - \tau _{i,\sigma }$), leading to temporal current fluctuations called shot noise^24^. The relation between shot noise and spin transmission channels for $eV \gg k_{\mathrm{B}}T$ is given by $S = 2eIF$, where *I* is the current and *F* is the Fano factor, defined as $F = \mathop {\sum }\nolimits_{i,\sigma } \tau _{i,\sigma }(1 - \tau _{i,\sigma })/\mathop {\sum }\nolimits_{i,\sigma } \tau _{i,\sigma }$. For an arbitrary number of channels *i* and spin type σ, the CSP depends on the Fano factor as^34^ $P_G \ge \left[ {(2G_0^{\mathrm{s}}(1 - F)/G) - 1} \right]^{1/2}$. Therefore, shot noise provides the lower bound for CSP. Figure 2a presents a plot of Fano factor as a function of conductance space, obtained by solving the above two equations for *F *and *G*. In the dark gray area, valid (*F,G*) solutions do not exist. Practically, (*F,G*) data located in this region would indicate an experimental artefact. In the light-gray area, only *F* and *G* combinations that lead to finite spin-polarization ($P_G \;> \; 0$) can exist^31–33^. The black lines indicate the minimal CSP for data that appear below it. For example, (*F*,*G*) data appearing below the line of 30% spin-polarization indicate $P_G\; > \; 30\%$ for the measured junction. Finally, data on the border of the dark gray region imply $P_G = 100\%$ (see Supplementary Notes 2 and 3).

In Fig. 2, the data measured for bare silver junctions (black dots) do not penetrate into the light-gray region within the measurement uncertainty (Supplementary Fig. 3), as expected for silver atomic junctions with spin degenerate conductance ($\tau _{i, \uparrow } = \tau _{i, \downarrow }$). Once vanadocene molecules are introduced to form molecular junctions, the distribution of the measured (*F,G*) data is significantly different, where the large data spread implies a rich set of possible molecular junction structures. Remarkably, a clear penetration to the light-gray region that indicates spin-polarized conductance is observed for many realizations of the Ag/vanadocene junctions. No penetration is observed at high conductance (*G* *>* 2 $G_0^{\mathrm{s}}$), because in these cases the junction is squeezed such that the contact is dominated by Ag atoms. Interestingly, some junctions feature a rather high CSP, as revealed by data below the *P*_*G*_ = 90% threshold line. While most of the examined junctions exhibit smaller CSP, these cases indicate that some of the molecular junction’s geometries act as a very efficient spin-filter.

It is important to stress that the revealed spin filtering in the examined non-magnetic molecular junction has both a concrete fundamental meaning and important practical advantages. From the fundamental point of view, the generated spin-polarized current in the examined non-magnetic system has a well-defined spin orientation in spin space, despite the absence of a defined orientation in the device frame of reference for spin-up and spin down. In this context, there is a fundamental difference between a current of both spin-up and spin down to a current dominated by only one spin type. Focusing on the practical aspects of our findings, the unique combination of spin filtering in the spin space and an undefined spin orientation with respect to the molecular conductor has interesting advantages. In magnetic systems, the injected spin direction is typically constrained and aligned with the magnetization of the system (e.g., the magnetization of ferromagnetic electrodes). In contrast, the spin current in the presented case has no preference in the device frame of reference. Therefore, it is sufficient to apply a minor magnetic field (${\mathbf{B}}\; > \; k_{\mathrm{B}}T/\mu$; *μ* is the magnetic permeability) to orient the spin direction of the spin-polarized current in a desired direction. This ability opens the door to very fast and flexible magnetic manipulations without the limitations imposed by magnetic material properties, including structural changes by magnetostriction, slow hysteretic switching, and power consumption that is translated to unwanted local heating. These advantages can be useful for fast spintronic operations and for the study of dynamical aspects of nanoscale magnetism and spin transport.

To shed light on the relation between our observations and the fact that vanadocene has a finite total spin, we performed control shot-noise measurements on Ag/ferrocene molecular junctions. We chose ferrocene because it has zero total spin and a structure similar to vanadocene, with an iron central atom. Figure 2b presents (*F*,*G*) data provided by shot noise and conductance measurements on a set of Ag/ferrocene molecular junctions (see conductance characterization in Supplementary Note 4 and Supplementary Fig. 5). In contrast to the case of Ag/vanadocene, here the (*F*,*G*) data do not penetrate into the light-gray area. Thus, for Ag/ferrocene junctions we do not find indications for spin-polarized conductance. These findings suggest that the finite spin of vanadocene plays a role in the generation of spin-polarized conductance.

**Fig. 3:**
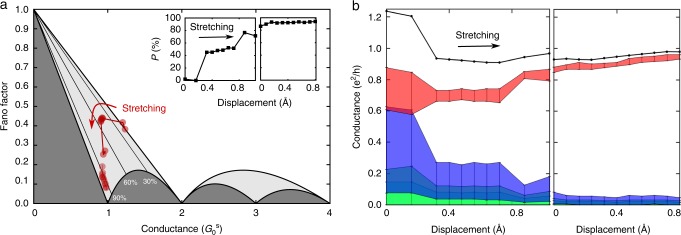
Experimental analysis of spin-polarized transport during elongation of Ag/vanadocene junctions. **a** Fano factor versus conductance during the elongation of two different Ag/vanadocene junctions. Following each measurement, the junction is stretched by 0.1–0.05 Å. The uncertainty, corresponding to systematic errors in our measurements, is comparable to the symbols’ diameter, as shown in Supplementary Fig. 4. Inset, lower bound for CSP (*P* is the minimal *P*_*G*_), as determined by the experimentally obtained Fano factor and conductance, with ±3% experimental uncertainty. **b** Total conductance (black dots, error range is given by dots’ diameter) and transmission probabilities of the largest four spin-polarized transmission channels (colors). The large uncertainty in the transmission probabilities, indicated by the error bars, stems from the numerical analysis (Supplementary Note 5). As the junction is stretched, the conductance evolves towards the upper limit of ballistic spin-polarized transmission, with conductance approaching $G_0^{\mathrm{s}}$, spin-polarization approaching 100%, and electron transport that is dominated by a single spin-polarized transmission channel (red-shaded). Here, the maximal values are: *G* *=* 0.98 ± 0.01 $G_0^{\mathrm{s}}$, *P*_*G*_ ≥ 95 ± 3%, with significant suppression of all spin channels except one. Additional examples for the evolution of transport properties as a function of junction elongation appear in Supplementary Fig. 7. See also Supplementary Note 6.

As seen in Fig. 2a, shot-noise measurements provide indications for relatively highly CSP for some realizations of Ag/vanadocene junctions. The evolution of spin-polarization and the distribution of spin channels for these molecular junctions can be examined during junction elongation. Figure 3 presents two sets of (*F*,*G*) data as a function of inter-electrode displacement, obtained for two different junction realizations. One set is recorded during the early stages of junction formation, revealing several transmission channels, and a second set shows conductance close to 1 $G_0^{\mathrm{s}}$ dominated by a single transmission channel. During the stretching process, the degree of minimal spin-polarization is increased up to 95 ± 3% (Fig. 3a, Inset). Figure 3b presents the corresponding evolution of the total conductance (black) and the contribution of each one of the four most dominant spin-polarized channels (colored) to the total conductance. The large uncertainty stems from analyzing numerically the contribution of more than two channels by using only two equations (for shot noise and conductance), as explained in Supplementary Note 5. When the junction has a total conductance of ~1.2 $G_0^{\mathrm{s}}$, it is carried by several spin-polarized channels. However, as the junction is stretched, most of the channels are suppressed and the conductance is dominated by a single spin-polarized transmission channel, which remains approximately fully-open. These conditions are unique in the sense that they are close to the characteristics of ideal ballistic spin-polarized transport. Namely, conductance of only one spin type with transmission probability of one.

### Calculations

**Fig. 4:**
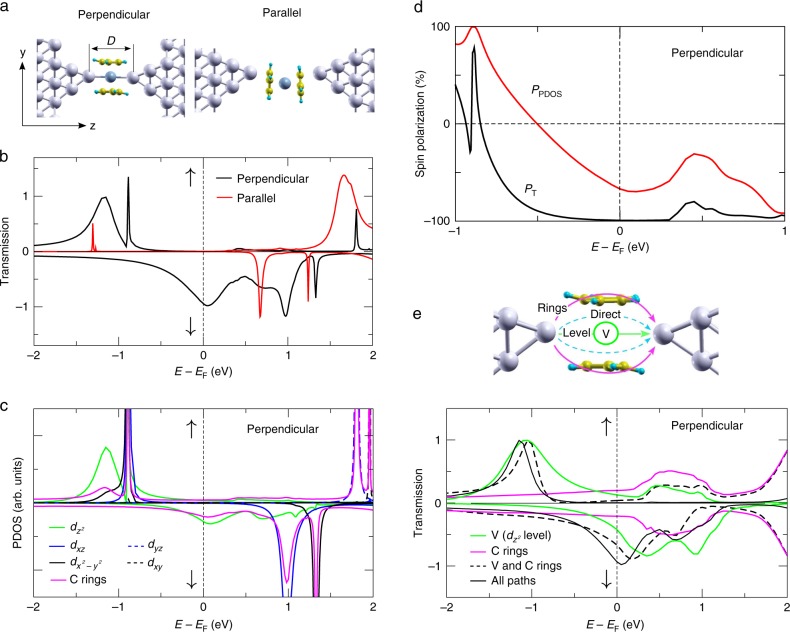
Calculations for spin-polarized transport across Ag/vanadocene junctions. **a** Two junction configurations characterized by perpendicular (left) and parallel (right) molecular orientation with respect to the junction axis. **b** Spin-resolved transmission versus energy for the two junction configurations. **c** Spin-resolved projected density of states (PDOS) on the vanadium *d* orbitals for perpendicular molecular configuration (see also Supplementary Note 10). The PDOS on the rest of the molecule (two carbon rings) is shown in pink. The origin for the different widths of the spin-up and down transmission peaks is discussed in Supplementary Note 11. **d** Spin-polarization of transmission and vanadium $d_{z^2}$ PDOS (*P*_*T*_ and *P*_PDOS_, respectively) for the perpendicular molecular configuration. Interestingly, *P*_*T*_ remains rather high even at *E* < −0.5 eV, where *P*_PDOS_ is nullified and even changes its sign. **e** Spin-resolved transmission for different current pathways across the junction in the perpendicular molecular configuration. Two main transmission pathways—across the carbon rings (pink lines) and across the vanadium $d_{z^2}$ level (green lines)—are found to play an essential role interfering destructively (constructively) at the Fermi level for spin-up (down) electrons, as seen by their overall contribution in black dashed lines. The black line describes all contributions (including direct silver-silver tunneling, for example). The origin for the shift in the transmission peaks is discussed in Supplementary Note 12.

To reveal the origin of the observed spin filtering by the Ag/vanadocene junction, we performed calculations of electronic structure and transport based on spin-polarized density-functional theory (DFT) (see Methods). Here, we focus on two junction structures, shown in Fig. 4a, which were found to be stable (see Supplementary Note 7 for structural analysis). Specifically, the perpendicular configuration (left) is more energetically favorable at smaller inter-electrode distances *D* (defined as the distance between the two Ag apex atoms), while for larger D the parallel configuration (right) is more stable (Supplementary Note 8).

As a first step, we compare the calculated transport properties of these junction structures with our experimental findings. Figure 4b shows spin-resolved total transmissions for two representative perpendicular (*D* = 5.8 Å; minimum total energy; Supplementary Note 9) and parallel (*D* = 8.6 Å) configurations. For the perpendicular configuration, the total transmission (up and down spins) is about 0.96 at the Fermi energy (giving a conductance of 0.96 $G_0^{\mathrm{s}}$), and is fully spin-polarized. In contrast, the junction with parallel molecular orientation has significantly lower total transmission at the Fermi energy, in the range of 10^–3^ and has very low spin-polarization. Generally, junctions with perpendicular orientation and different inter-electrode separations have a typical total calculated transmission in the range of 0.2–1 at the Fermi energy and a very high spin-polarization (Supplementary Note 9). The agreement of these findings with the experimental indications for spin-polarized conductance in the range of 0.2–1 $G_0^{\mathrm{s}}$ suggest that junctions with perpendicular molecule orientation (Fig. 4a, left) are responsible for the experimentally detected spin-filtering. Supplementary Fig. 6 reveals, on top of significant conductance contribution around 1 $G_0^{\mathrm{s}}$, lower characteristic conductance with a large spread around 10^−2^–10^−3^ $G_0^{\mathrm{s}}$, which can be ascribed to the parallel molecular orientation.

At first sight, the mechanism behind the strong spin filtering found for the perpendicular Ag/vanadocene junctions seems to be rather straightforward. Figure 4c presents the spin-resolved projected density of states (PDOS) on the vanadium *d*-orbitals, which dominate the molecular states around the Fermi level (for completeness, the pink line in Fig. 4c shows the PDOS on the rest of the molecule). These *d*-originated states are split due to ligand and magnetic exchange fields, and are mostly represented at the Fermi energy by the $d_{z^2}$ orbital (Fig. 4c, green lines).

Due to symmetry and spatial orientation, these orbitals hybridize strongly with the silver *s*-states and therefore provide the main transport channel. Consequently, the spin-dependent transmissions follow very closely their shape (superposing with some sharp features due to other molecular orbitals weakly coupled to the electrodes’ states, e.g., at *E* = −0.85 eV for spin-up). Due to rather different spin-up and spin down $d_{z^2}$ PDOS at the Fermi energy, different conductance for each spin type is expected. To quantify the effect, Fig. 4d shows the calculated spin-polarization of $d_{z^2}$ PDOS as a function of energy, defined as $P_{{\mathrm{PDOS}}}\left( E \right) =\left[ {{\mathrm{PDOS}}^ \uparrow \left( {\mathrm{E}} \right) - {\mathrm{PDOS}}^ \downarrow \left( {\mathrm{E}} \right)} \right]/\left[ {{\mathrm{PDOS}}^ \uparrow \left( {\mathrm{E}} \right) + {\mathrm{PDOS}}^ \downarrow \left( {\mathrm{E}} \right)} \right]$, where ${\mathrm{PDOS}}^{\upsigma}\left( {\mathrm{E}} \right)$ is the spin-resolved PDOS for spin-up and down. At the Fermi energy the resulting spin-polarization is only $P_{{\mathrm{PDOS}}}\left( {E_F} \right) = 67\%$, which is substantial but still not large enough to explain the extreme cases, for example when CSP above 90% is detected by shot-noise measurements (Fig. 2a). Interestingly, previous measurements of transport across cobalt and iron atoms adsorbed between gold electrodes showed limited CSP (30–60%) that is rather typical of a spin-polarized density of state^20^. In contrast, the calculated transmission spin-polarization (Fig. 4d, black curve), defined as $P_T\left( E \right) = \left[ {T^ \uparrow \left( E \right) - T^ \downarrow \left( E \right)} \right]/\left[ {T^ \uparrow \left( E \right) + T^ \downarrow \left( E \right)} \right]$ for a total spin-resolved transmission $T^{\upsigma}$, is nearly -100%, over a wide range of energies around the Fermi energy. Here, minus indicates a dominant spin-down contribution. The calculated *P*_*T*_$\left( {E_{\mathrm{F}}} \right)$ describes well the experimental indications for high CSP in some of the examined junction configurations. However, our calculations reveal that the high transport spin polarization cannot be escribed to differences in the density of states for opposite spins. We note that further calculations using different methodology (Supplementary Note 8 and Supplementary Fig. 11) provide similar results.

## Discussion

In what follows, we present clear indications that the very high spin-polarization found both by experiment and transmission calculations stems from quantum interference between different current pathways across the Ag/vanadocene junction. The signature of interference is seen in the transmission calculations (Fig. 4b) but not in the PDOS calculations (Fig. 4c) because it is an outcome of interfering electronic transport pathways, rather than an outcome of local orbital hybridization. The first hint for interference is the asymmetric shape of the rather broad spin-up transmission peak centered at around E = −1.2 eV (Fig. 4b). While the transmission reduction is moderate at *E* < −1.2 eV, it is severely suppressed towards the Fermi energy, where it is negligibly small (0.005), giving rise to essentially $P_T(E_F) \cong 100\%$. In contrast, the PDOS seen in Fig. 4b remains finite, leading to $P_{{\mathrm{PDOS}}}(E_{\mathrm{F}}) = 67\%$ (Fig. 4d). The interference in transmission is revealed upon considering the contributions of different pathways to the total transmission of the examined junction, seen in Fig. 4e, which can be easily calculated within our tight-binding code by setting appropriate hopping parameters to zero. For spin-up transmission, the individual contributions across the vanadium $d_{z^2}$ level (green) and the *π* system of the carbon rings (pink) are both significant near the Fermi energy, but the total contribution of the two pathways (black dashed) is clearly lower than their individual contributions. This indicates a destructive interference near the Fermi energy. Additional secondary pathways (e.g., direct tunneling between the electrodes) further contribute to the destructive interference, leading to yet more efficient suppression in the total spin-up transmission (black). The interplay between the different pathways for spin-down is considerably different. In this case, the two main pathways (as well as all pathways) contribute constructively at the Fermi energy, summing up to a transmission almost equal to one, and hence to highly spin-polarized conductance close to 1 $G_0^{\mathrm{s}}$.

The demonstrated spin-dependent quantum interference is essential for having highly spin-polarized conductance in the examined molecular junction. This mechanism stems from an interplay of two main effects, as can be seen in Fig. 4b: an asymmetric shape of the $d_{z^2}$ related transmission peak (as can be clearly observed for spin-up channel around −1.2 eV), reflecting the destructive (constructive) interference to the right (left) of its maximum; and a single occupancy of the highly conductive vanadium $d_{z^2}$ level, which therefore appears below (above) the Fermi energy for spin-up (spin-down) electrons. While the effect of quantum interference on charge transport has been extensively studied experimentally in molecular junctions^11–19^, the effect of quantum interference on spin transport has not been demonstrated in such junctions or, generally, at the nanoscale. Specifically, spin-dependent quantum interference was studied experimentally merely in large systems, such as microscale GaAs and graphene devices^34,35^, which are more sensitive to decoherence effects due to their size. The presented spin-dependent quantum interference in molecular junctions can be nicely reproduced by a tight-binding toy model as discussed in Supplementary Note 13, where it is also shown how the resulting transmission line-shapes can be readily rationalized in terms of scattering phase shifts.

To conclude, we experimentally demonstrate that a single-molecule junction can act as an efficient spin-filter without the use of ferromagnetic components. The conductance of the examined Ag/vanadocene molecular junctions approaches in some cases the limit of ideal ballistic spin transport, where it is primarily dominated by a single spin-polarized transmission channel, which is nearly fully open. Such efficient spin filtering in a non-magnetic nanoscale system pave the way to fast and power-saving spin-current manipulations based on small and dynamic magnetic fields. Based on detailed comparison of ab initio calculations to shot-noise and conductance measurements, we ascribe the efficient spin filtering found for many of the examined junctions to spin-dependent quantum interference. This mechanism is based on the combination of spin-split energy levels near the Fermi energy and quantum interference. Together, they lead to destructive interference for one spin type and constructive interference for the opposite spin type at the Fermi energy. Our findings show that spin-dependent quantum interference can be obtained at the single-molecule level. This mechanism can be used as an efficient scheme for spin filtering at the nanometer down to sub-nanometer scale, with the advantage of lower sensitivity to decoherence effects in these length scales.

## Methods

### Experiments

Our experiments were conducted in a mechanically controllable break-junction^11^ setup (MCBJ) placed in a liquid helium cryostat. A silver wire (99.997%, 0.1 mm, Alfa Aesar) with a notch in its center is attached to a flexible substrate (1-mm-thick phosphor-bronze covered by 100 μm insulating Kapton film). This structure is placed in a cryogenic insert, which is pumped to 10^−4^ mbar and then cooled down to 4.2 K by dipping it in the cryostat. A three-point bending mechanism is used to bend the substrate (Fig. 1b). Consequentially, the wire is broken at the notch, exposing (in cryogenic vacuum) two ultra-clean atomically sharp tips that are used as the junction’s electrodes. A piezoelectric element (PI P-882 PICMA) is used to tune the bending of the substrate and control the inter-electrode distance with sub-Å resolution. This element is driven by a 24-bit NI-PCI4461 data acquisition (DAQ) card, followed by a Piezomechanik SVR 150/1 piezo driver. An ensemble of junctions with diverse structures is studied by repeatedly pushing the electrodes together to form a contact of ~100–140 $G_0^{\mathrm{s}}$ and pulling the electrodes apart until full rupture. The vanadocene or ferrocene molecules (95%, Stream Chemicals and 99.5%, Alpha Aesar, respectively, further purified in situ) are introduced into the metallic junction by sublimation from a locally heated molecular source towards the repeatedly broken and reformed junction^12^. In order to measure the conductance traces, direct-current (d.c.) conductance is probed, when the junction is gradually broken by increasing the applied voltage on the piezoelectric element at a speed of 600 nm s^−1^ and a 100 kHz sampling rate. A constant voltage of 10–200 mV is applied across the junction by a NI-PCI4461 DAQ card. The generated current is amplified by a current preamplifier (Femto DLPCA-200) and recorded by the DAQ card. The obtained conductance is given by the measured current divided by the applied voltage. The inter-electrode displacement is estimated by the dependence of tunneling current on the distance between the electrodes. Measurements of differential conductance (*dI/dV* versus *V;* e.g., Supplementary Fig. 2) are performed using a standard lock-in technique. A reference sine signal of 1 mV peak-to-peak voltage (*V*pp) at ~3 kHz, modulating a d.c. bias voltage is supplied by the DAQ card. The alternating-current (a.c.) response is recorded by the DAQ card and extracted by either a LabView implemented lock-in or a SR830 lock-in to obtain the differential conductance as a function of applied voltage. Noise measurement are performed when the instruments used for conductance measurements are disconnected from the sample to reduce extrinsic noise. The voltage noise generated in the junction is amplified by a custom-made differential low-noise amplifier, which is calibrated by the thermal noise of a set of known resistors located in liquid nitrogen. A power spectrum between 0.25 kHz and 300 kHz is measured via the NI-PXI5922 DAQ card, using a LabView implemented fast Fourier transform analysis and averaged 3,000 times. To probe shot noise, the sample is biased by current, using a voltage source (NI-PCI4461 DAQ card) connected to the sample through two 2 MΩ resistors located near the sample (the resistors convert the voltage bias into current bias). All amplifiers are powered by batteries to avoid the injection of unwanted noise from power lines.

### Calculations

Spin-polarized density-functional theory (DFT) calculations shown in Fig. 4 were performed using planewaves and ultrasoft pseudopotentials, as implemented in the Quantum-ESPRESSO (QE)^36^ package. The Perdew-Burke-Ernzerhof (PBE)^37^ exchange-correlation functional was used throughout. The molecular junctions shown in Fig. 4 were described by a supercell containing the molecule and two 4-atom Ag pyramids attached to a Ag(111) slab containing five and four atomic layers on the left and right side, respectively. A 4 × 4 in plane periodicity (16 atoms per Ag layer) was employed to avoid artificial molecule/molecule interactions. Spin-polarized transport was studied using a home-made tight-binding code^38^ with tight-binding parameters extracted from the first principles QE calculations. The standard non-equilibrium Green's function (NEGF) formalism was employed to compute spin-dependent transmission functions, defining the coherent conductance in the framework of the Landauer-Büttiker formalism.

The calculations presented in Supplementary Fig. 10 and 11 were performed as follows. The molecular geometry of the junction was the same as in the above calculations. Electronic structure calculations were carried out using density-functional theory (DFT) as implemented in the SIESTA^39^ and VASP^40^ codes, with the PBE exchange-correlation functional. Geometrical optimization was performed using VASP and the optimized geometries were used in the SIESTA calculations. Spin-dependent electron transport calculations were performed using the TRANSIESTA^41^ code, which performs a non-equilibrium Green’s-function (NEGF) calculation based on the DFT results.

## Supplementary information

Supplementary Information

## Data Availability

The data that support the plots within this paper and other findings of this study are available from O.T., A.S., and L.K. upon reasonable request.
