# Supplementary material for: Nonmagnetic single-molecule spin-filter based on quantum interference
Source: Nat Commun. 2019 Dec 5;10:5565. doi: 10.1038/s41467-019-13537-z (PMC6895237; doi:10.1038/s41467-019-13537-z)
Supplement: Supplementary file 1 — Supplementary Information [file 41467_2019_13537_MOESM1_ESM.pdf]

SUPPLEMENTARY INFORMATION

Nonmagnetic single-molecule spin-filter based on quantum interference

Pal *et al*.

Supplementary Note 1.

Electronic measurement circuit

Supplementary Fig. 1 shows the electronic setup connected to the sample. The circuit can be switched between a Conductance Mode, which is used to measure d.c. conductance as well as a.c. (differential) conductance spectra, and a Noise Mode, in which thermal noise and shot noise are measured. In the latter mode of measurement, the relatively noisy instruments used in the conductance mode are disconnected from the sample because of the high sensitivity of the noise measurements.

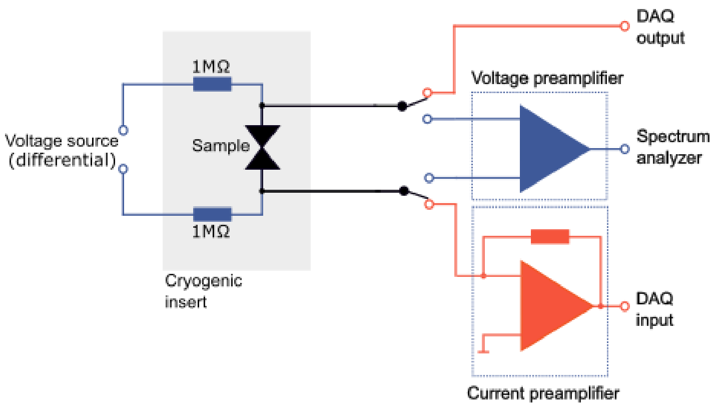

Supplementary Figure 1. Electronic circuit of the measurement setup. Schematic presentation of the electronic circuit for conductance and noise measurements. Two switchable measurement circuits are observed: a conductance circuit (orange) and a noise circuit (blue). The section cooled to 4.2 K is marked in light gray.

Supplementary Note 2.

Shot noise measurements

The overall zero-frequency current noise (both thermal and shot noises) generated in a quantum coherent conductor can be expressed in the framework of Landauer formalism as[1,2]

$$S_I = 4k_BTG[1 + F(x\coth(x) - 1)], \qquad (1)$$

where $x = eV/2k_BT$ provides the ratio between an applied voltage V and temperature T. At near-equilibrium conditions (x≪1), equation (1) reduces to the thermal noise expression[1] $S_I = 4k_BTG$. At high applied voltage (x≫1), the current noise depends linearly on the current, $S_I = 2eIF$. Supplementary Fig. 2 presents an example of shot noise analysis in an Ag/vanadocene molecular junction. Before and after each set of noise measurements as a function of bias voltage, differential conductance spectra (*dI/dV* versus *V*; Supplementary Fig. 2a), are recorded in order to confirm that the junction has remained stable during the noise measurements. The zero-bias conductance of the junction is determined from the average differential conductance in a window of $|V| \leq 5\ mV$. Variations in the conductance at this range are taken into consideration as an experimental error.

Supplementary Fig. 2b presents a set of noise spectra for different applied current. Note that the bias current produces bias voltage across the junction ($V = I/G$). Each spectrum is obtained from the Fourier transform of voltage fluctuations produced by the junction, and averaged for 3,000 consecutive measurements. The voltage noise produced by the amplifier was measured separately and subtracted from the recorded spectra. The measured voltage noise is presented as a current noise using the relation $S_I = S_V \cdot G^2$. Next, the spectra were corrected to account for low-pass RC filtering due to the resistance of the sample and cabling, as well as the amplifier input capacitance and the capacitance of the cabling (total capacitance of $\sim$40 *pF*). The noise power is averaged in a frequency window, in which $1/f$ noise contributions are negligible (marked by red lined in Supplementary Fig. 2b). Supplementary Fig. 2c shows the obtained average current noise power as a function of the generated bias voltage across the junction. Following Ref. 2, Eq. (1) can be expressed as

$$Y = F(X - 1). \qquad (2)$$

Here, $X = x coth(x)$ and $Y = [S_I(V) - S_I(0)]/S_I(0)$. The Fano factor is obtained by calculating the reduced parameters X and Y and obtaining a linear fit of $Y(X)$ according to Eq. (2), as shown in Supplementary Fig. 2d. Occasionally, we observed zero-bias anomalies in the differential conductance curves that may originate from different possible mechanisms, including Kondo physics. The data obtained in these specific cases will be analyzed and reported elsewhere, since the physics involved is beyond the Landauer formalism considered here.

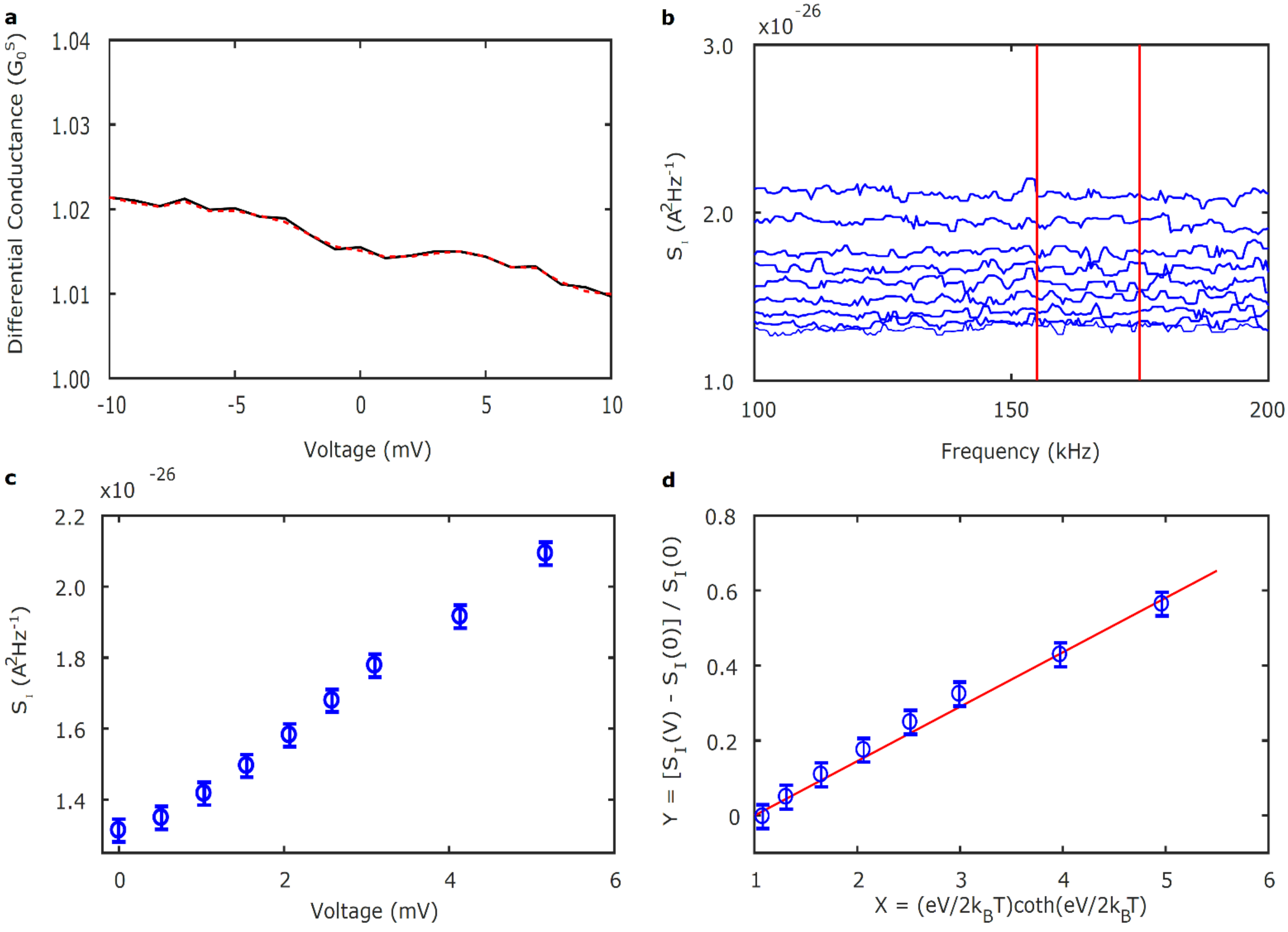

Supplementary Figure 2: Analysis of shot noise measured in an Ag/vanadocene junction. a, Differential conductance versus voltage curves measured for an Ag/vanadocene junction before (black) and after (dashed red) noise measurements. **b,** A series of current noise spectra for different bias voltage across the junction. Red lines indicate the frequency window that is selected to obtain the average noise power. **c,** Average current noise power as function of bias voltage calculated using the presented noise in (b). **d,** Reduced parameter Y as a function of X (blue) calculated using the measured noise in (c), and a linear fit (red), giving $F = 0.15 \pm 0.01$ according to Supplementary Eq. (2).

Supplementary Note 3.

Determination of conductance spin polarization by shot noise measurements

Shot noise measurements were used in a variety of nanoscale and mesoscopic systems to gain information on spin transport effects[2-7]. In a quantum coherent conductor with an unknown number of transmission channels, the combination of conductance and shot noise measurements provides a lower bound for the degree of conductance spin polarization (CSP)[6]. In the following, we wish to elaborate on the meaning of the (F, G) plot presented in Fig. 2 and the information that can be derived from this plot. In the Landauer framework, shot

noise depends on $\sum_{i,\sigma} \tau_{i,\sigma} (1 - \tau_{i,\sigma})$, whereas the conductance depends on $\sum_{i,\sigma} \tau_{i,\sigma}$. The (F, G) plot illustrates this dependence.

For zero spin-polarization, the conductance is carried by one or more pairs of spin-polarized transmission channels for opposite spins with equal transmissions ($\tau_{1,\uparrow} = \tau_{1,\downarrow}, \tau_{2,\uparrow} = \tau_{2,\downarrow}, \ldots$). For example, the conductance of a single-atom junction of silver is carried by two equal and opposite spin-polarized transmission channels that are associated with the single *s* valence orbital of silver. For zero CSP, the (F, G) data points can have any value in the white region of Fig. 2 in the main text. However, the dark and light gray regions are forbidden. For finite spin-polarization, the constraint $\tau_{i,\uparrow} = \tau_{i,\downarrow}$ does not hold and the forbidden region (presented now in dark gray) is "squeezed" by a factor of 2 along the conductance axis. As a result, any (F, G) combination that is located in the light gray region indicates a finite spin-polarization.

A lower bound for the CSP can be given by the following analysis. Assuming only two spin-polarized channels of opposite spin type, the Fano factor can be rewritten in terms of P_G^{2ch} (P_G is defined in the main text, and P_G^{2ch} is P_G for two channels) as follows: $F = 1 - G(1 + (P_G^{\mathrm{2ch}})^2)/2G_0^{\mathrm{s}}$, and hence $P_G^{\mathrm{2ch}} = [2G_0^{\mathrm{s}}/(1 - F)/G - 1]^{1/2}$. In contrast, for two spin-polarized channels of the same spin type, the CSP is 100%. Since shot noise does not provide information about the spin type, for two spin channels of an unknown spin type the CSP is either P_G^{2ch} or 100%. According to the derivation that appears in the Supplementary Information of Ref. 6, for any number of spin-polarized transmission channels $P_G \geq P_G^{\mathrm{2ch}}$ holds. As a result, shot noise measurements (accompanied with conductance measurements) provide the lower bound of CSP for an arbitrary number of spin-polarized transmission channels. In the special case of conductance carried by a single spin-polarized transmission channel, (F, G) data are located on the border of the dark gray region in Fig. 2 (thick black line), where P_G = 100%.

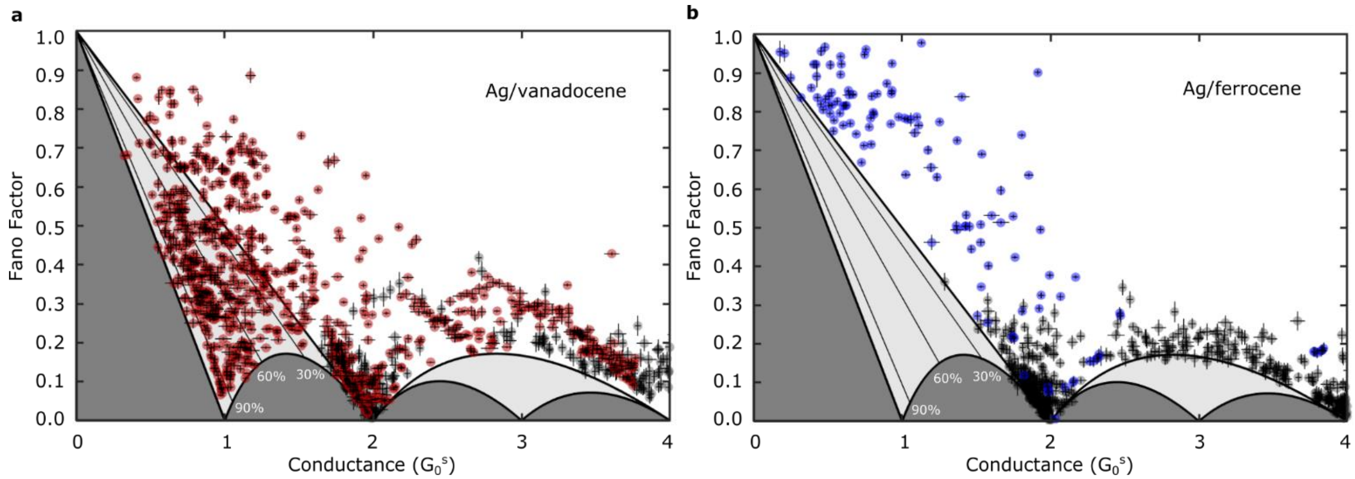

Supplementary Figure 3: Fano Factor for bare silver atomic scale junctions, Ag/vanadocene (*S*=3/2) and Ag/ferrocene (*S*=0) molecular junctions. Fano factor (F) extracted from shot noise and conductance measurements on the examined junctions as a function of conductance (G), as presented in Fig. 2a, b, including error bars, corresponding to the systematic errors in our measurements. The size of the error bars is comparable to or slightly larger than the diameter of the semitransparent symbols.

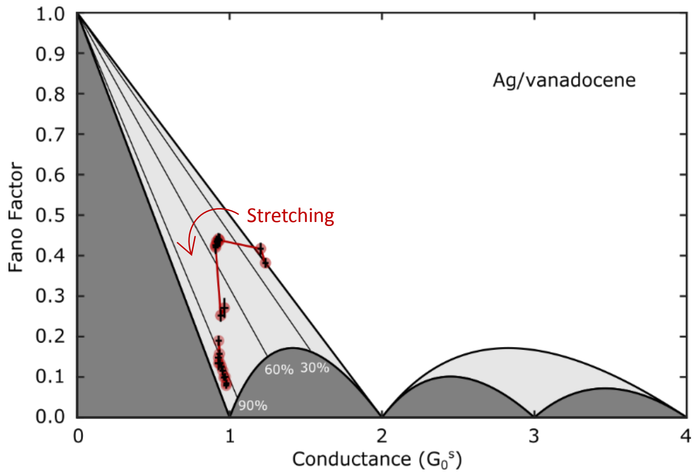

Supplementary Figure 4: Fano Factor during elongation of Ag/vanadocene molecular junctions. Fano factor extracted from shot noise and conductance measurements of the examined junctions versus conductance, during the elongation of the junctions as presented in Fig. 3a, including error bars. The error bars correspond to systematic errors in our measurements. The size of the error bars is comparable to the diameter of the semitransparent symbols.

Supplementary Note 4.

Conductance measurements of Ag/ferrocene and Ag/vanadocene molecular junctions

In Supplementary Fig. 5a,b, left panels, plateaus and tilted plateaus below the peak of 2 G_0^S and above 0.1 G_0^S can be observed. These conductance values are repeatable despite the fact that 2-5·10^{-2} G_0^S is the most probable conductance of Ag/ferrocene junctions as seen in Supplementary Fig. 5c,d, middle panels. Based on comparison of the conductance histograms in Supplementary Fig. 5a,b, middle panels, it is clear that the contribution of these plateaus to the histogram is higher during the formation process (Supplementary Fig. 5b; i.e., they are more abundant during this process). In view of these observations, the shot noise measurements that are performed on Ag/ferrocene junctions and presented in Fig. 2b were taken on junctions with different fixed inter electrode distance, following a formation process. During this procedure, the opposite electrodes are brought together until a contact is formed. Repeating this procedure provides shot noise measurements of different junction configurations characterized by different conductance.

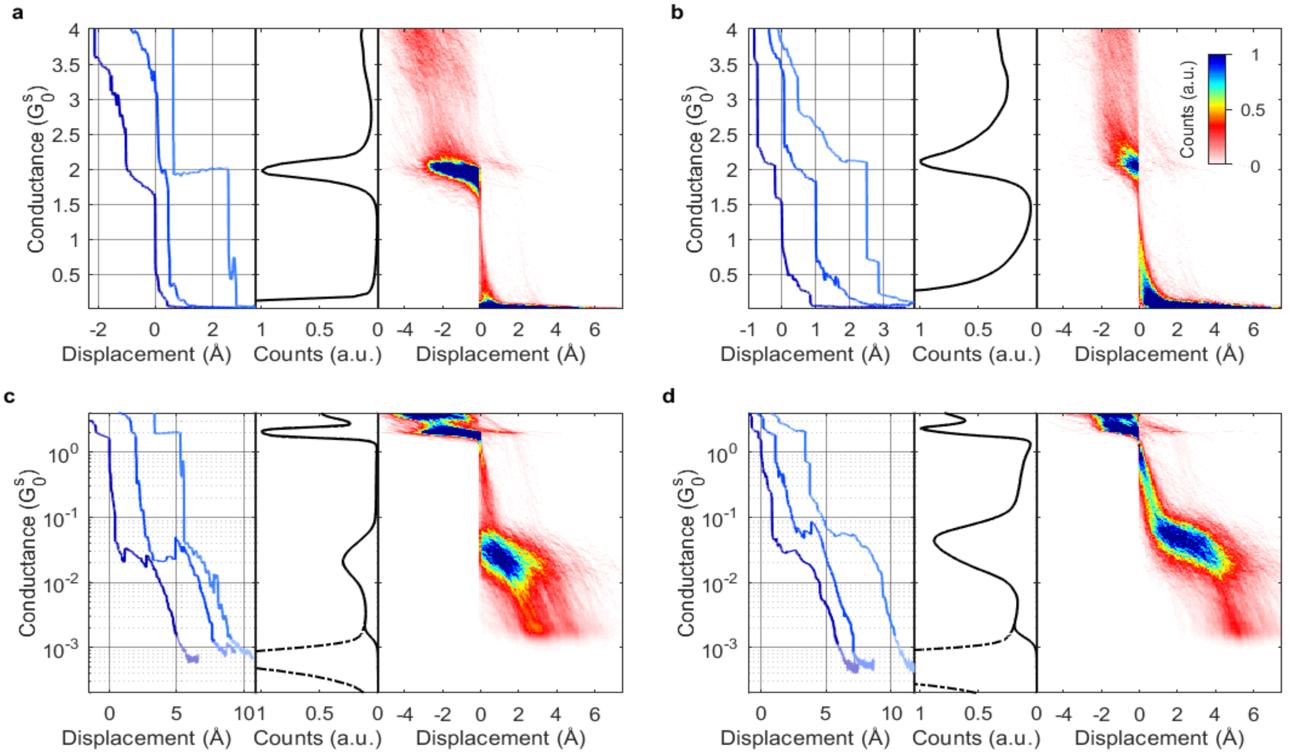

Supplementary Figure 5: Conductance of Ag/ferrocene molecular junctions. Characterization of Ag/ferrocene molecular junctions, where the data are presented in a linear (a,b) and a logarithmic (c,d) conductance scale, with data measured during junction breaking (a,c) and reformation (b,d). In each figure: **Left panel**, examples for conductance versus inter-electrode displacement traces. Traces are shifted in the displacement axis for clarity. **Middle panel**, conductance histogram constructed from 2,000 conductance traces recorded at a bias voltage of 100 mV. **Right panel**, conductance–displacement density plot based on the same conductance traces. Zero displacement is set for each trace as the first displacement point with a conductance below 4 G_0^S.

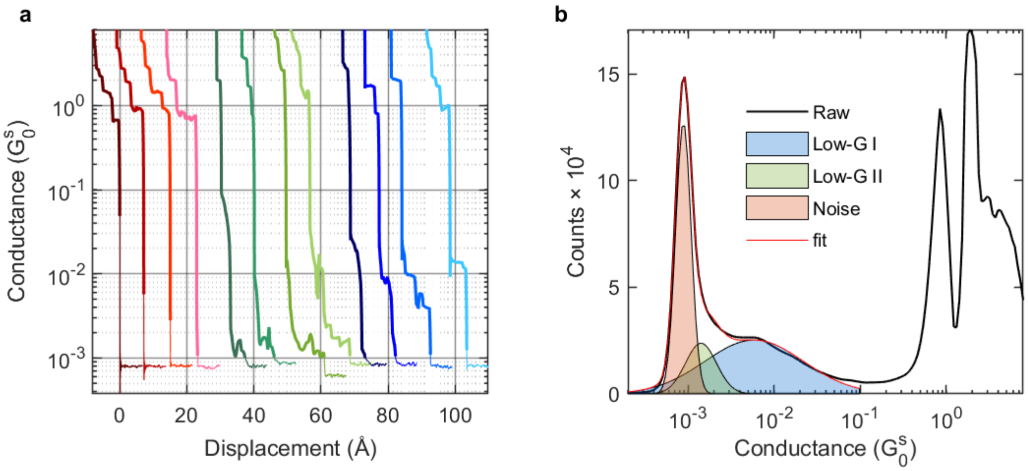

Supplementary Figure 6: Conductance of Ag/vanadocene molecular junctions. a, Examples for conductance as a function of inter-electrode displacement traces, recorded at a bias voltage of 100 mV. The traces are shifted in the displacement axis for clarity. Here, the plateau around 1 G_0^s is sometimes followed by another plateau with a lower conductance around 10^{-2}-10^{-3} G_0^s, at larger inter-electrode distances. **b,** Conductance histogram constructed from more than 2,000 traces. The histogram can be fitted (red curve) at conductance below 10^{-1} G_0^s to three Gaussians. The red Gaussian describes the amplifier noise floor and the others reveal two main conductance distributions, centered at $6{\cdot}10^{-2}$ G_0^s (Low-G I; blue Gaussian) and $1.4{\cdot}10^{-3}$ G_0^s (Low-G II; green Gaussian). Examples for the traces that construct each Gaussian are given in (a), using a color code.

Supplementary Note 5.

Numerical analysis of transmission channel distributions

We briefly describe the numerical procedure used to determine the channel distribution in Fig. 3b in the main text. The full derivation can be found in Ref. 8. A combination of F and G extracted from shot noise and conductance measurements can result from an infinite number of possible combinations of transmission probabilities, $\tau_{i,\sigma}$. However, one can limit the number of possible transmission sets to a finite number by using a finite precision $\Delta\tau$ for the transmission probabilities. For a given number of transmission channels 2N (N channels of each spin type), our procedure enumerates the $2N/\Delta\tau$ possible sets of transmission probabilities, $\{\tau_{i,\sigma}\}_{i=1}^{N}$. We can set the coefficients to be ordered by decreasing transmission: $\tau_{1,\sigma} \geq \tau_{2,\sigma} \geq \cdots \tau_{N,\sigma}$. Note that the difference between adjacent transmission coefficients is $\Delta\tau$. For each set, the Fano factor and conductance are computed and compared to the experimental values of F and G. A set of transmission probabilities is considered to match the experimentally based (F, G) values if it satisfies the following inequalities:

$$G - \Delta G \leq G_0^s \sum_{\sigma,i=1}^{i=N} \tau_{i,\sigma} \leq G + \Delta G, \qquad (3)$$

$$F - \Delta F \leq \sum_{\sigma,i=1}^{i=N} \tau_{i,\sigma}(1-\tau_{i,\sigma}) / \sum_{\sigma,i=1}^{i=N} \tau_{i,\sigma} \leq F + \Delta F\ , \qquad (4)$$

where ΔG and ΔF are the experimental errors in G and F, respectively. We define $\{\tau_{j,i,\sigma}\}_{i=1}^{N}$ as the jth set out of k transmission sets that match the experimental values. The transmission coefficient $\tau_{\mathrm{i},\sigma}$ can now be determined to be in the range between $\tau_{j,i,\sigma}^{\min} = \min\{\tau_{\mathrm{i},\sigma}\}_{j=1}^{k} - \Delta\tau$ and $\tau_{j,i,\sigma}^{\max} = max\{\tau_{\mathrm{i},\sigma}\}_{j=1}^{k} + \Delta\tau$. Here, $\Delta\tau$ are added to certify that all possible solutions for $\tau_{i,\sigma}$ are included in this range. In this analysis, we assume *a priori* a given number of channels. However, we can repeat the procedure for *N*+1, *N*+2,… to obtain convergence of the channel distribution. This procedure helps us avoid a deficient estimation of N.

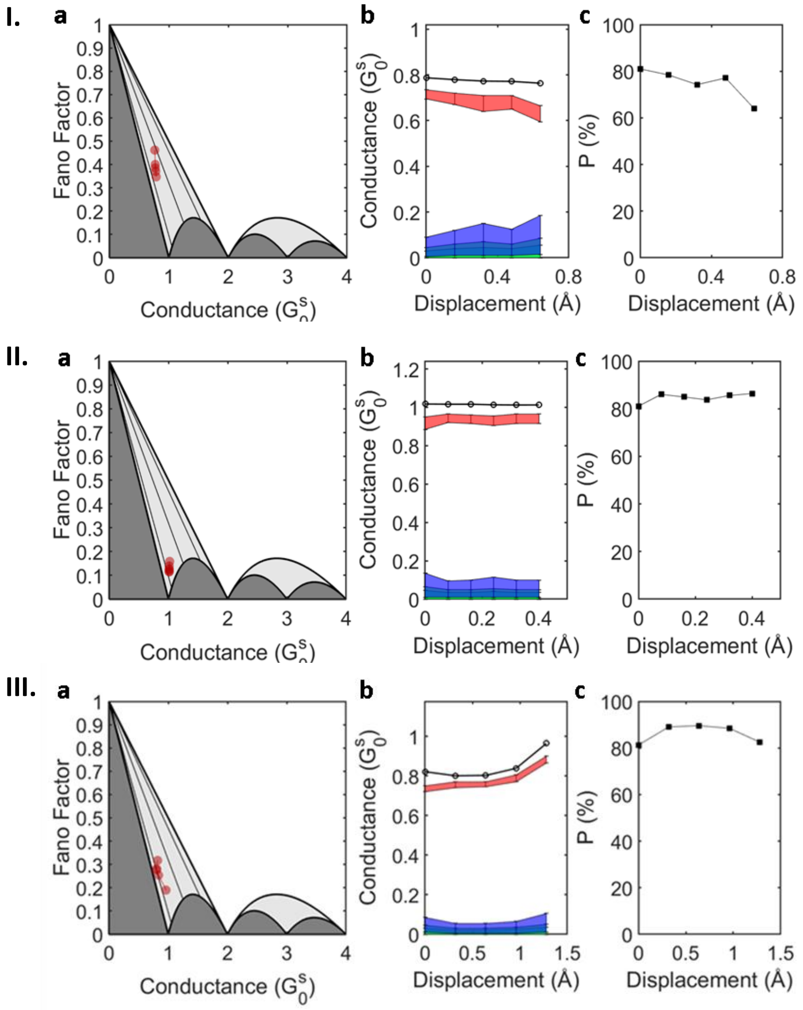

Supplementary Figure 7: Experimental analysis of spin-polarized transport during elongation of Ag\vanadocene junctions, as in Fig. 3 in the main text. **a,** Fano factor versus conductance during the elongation of three different Ag\vanadocene junctions. Following each measurement, the junction is stretched by up to 0.25 Å. The uncertainty, corresponding to systematic errors in our measurements, is comparable to the diameter of the semitransparent symbols, as shown in Supplementary Fig. 3 and 4. **b**, Total conductance (black dots, error range is given by dots' diameter) and transmission probabilities of the largest four spin-polarized transmission channels (colors). The large uncertainty in the channel transmissions stems from the numerical analysis (Supplementary Note 5). **c**, lower bound for CSP (P is the minimal P_G), as determined by the experimentally obtained Fano factor and conductance, with ± 3% experimental uncertainty. The different panels exemplify reduction (I), insensitivity (II), and non-monotonic (III) CSP response to junction stretching.

Supplementary Note 6.

Experimental analysis of spin-polarized transport during elongation of Ag/vanadocene junctions

The goal of Fig. 3 in the main text is to show that in some cases the conductance can approach the limit of ideal spin polarized ballistic conductance. The limit is given by a single fully open spin polarized transmission channel, namely $G=e^2/h$, $F=0$. As a result, we focus in Fig. 3, main text on stretching sequences, approaching this limit. Examining the entire measured data of transport vs. stretching, including cases that do not approach the spin-transport ballistic limit, we find that the CSP can evolve in different ways in response to junction elongation. This is actually expected considering the non-monotonic evolution of spin polarization as a function of inter-electrode distance as revealed by the different DFT and transport calculations presented in Supplementary Fig. 11 and 12. Over all, we examined 43 shot noise vs. junction elongation traces (see some example in Supplementary Fig. 7) and found 11 traces with increased CSP, 8 traces with decreased CSP, 16 traces with a non-monotonic behavior, and 8 traces with CSP rather insensitive to stretching. Since not always a single trace can capture the entire non-monotonic behavior, these results are in line with the outcome of our calculations.

Supplementary Note 7.

Structural analysis based on DFT and transport calculations

The connection of the vanadocene molecule to the electrodes is based on chemical binding (see further analysis of the total binding energy in Supplementary Note 9). Therefore, the number of stable junction configurations is limited to molecular orientations that allow such binding, and can be classified into several archetypical configurations. The classification describes the different symmetries possible for the chemical binding between the molecule and the electrodes.

Supplementary Figure 8 presents five different architypes of molecular junction configurations, with different chemical binding sites. For convenience, we also present the perpendicular (in-axis) and parallel (off-axis) molecular orientation (Supplementary Fig. 8a,b) considered in the main text, and we add the following configurations: parallel in-axis (Supplementary Fig. 8c), tilted (Supplementary Fig. 8d), and perpendicular off-axis (Supplementary Fig. 8e). Interestingly, when the structures are allowed to energetically relax, both the parallel in-axis molecular configuration (Supplementary Fig. 8c) and the tilted configuration (Supplementary Fig. 8d) relax to parallel off-axis orientation (Supplementary Fig. 8b), indicating that these two configurations are not energetically stable. When the perpendicular off-axis configuration (Supplementary Fig. 8e) is allowed to relax, the molecular junction breaks and the molecule is adsorbed on the facet of one of the electrodes, pointing that also this configuration is instable. These observations reveal that out of the different binding possibilities

between the molecule and the electrodes considered here, only the parallel off-axis and perpendicular in-axis configurations (Supplementary Fig. 8a,b) are energetically stable. We further note that the calculated conductance and conductance spin polarization (CSP) for the configurations presented in Supplementary Fig. 8c-e deviate considerably from the experimental observations of interest, where the conductance is around $1G_0^s$ and the CSP is high. In contrast, only the perpendicular (in-axis) configuration reveals conductance and CSP that match well the mentioned experimental observations. In view of the stability of the different configurations and their transport properties, we conclude that the perpendicular in-axis and parallel off-axis configurations (denoted in the main text as perpendicular and parallel configurations for convenience) can be assigned to the experimentally found most probable high and low conductance cases, respectively. Furthermore, based on transport properties, only the perpendicular in-axis configuration can be associated with the measured high spin filtering.

Focusing on the perpendicular in-axis molecular orientation that is associated with high spin filtering, we now examine the influence of the electrode structure on the conductance (i.e., the transmission at the Fermi energy in G_0^s units) and conductance spin polarization. As seen in Supplementary Fig. 9, we find that it is enough to have one flat electrode or even one diatomic tip to lose the spin filtering effect and have a significantly lower conductance than in the experimentally observed cases of interest. This is simply due to the lack of direct binding between the vanadium atom and a protruding silver atom on each of the two electrodes that is required in order to have significant electronic transport via this pathway.

Based on the above comparison between the calculations described here and the experimental results, we conclude that the ensemble of molecular junctions that show high conductance and high spin filtering in the experiments should have the following typical structural properties: (i) perpendicular in-axis molecular orientation; and (ii) direct binding between the vanadium atom and a protruding atom on each silver electrode. The abundance of high spin filtering (for example, CSP<70%) found in the experiments together with high conductance suggests that these structural properties of the Ag/vanadocene junction are not very rare. As discussed in the main text, the significant spin filtering found for the calculated perpendicular molecular junction configuration is an outcome of spin dependent quantum interference. We note that the examination of additional archetypical structures of molecular junctions does not reveal an alternative explanation for the high spin filtering found in some of the measured molecular junctions.

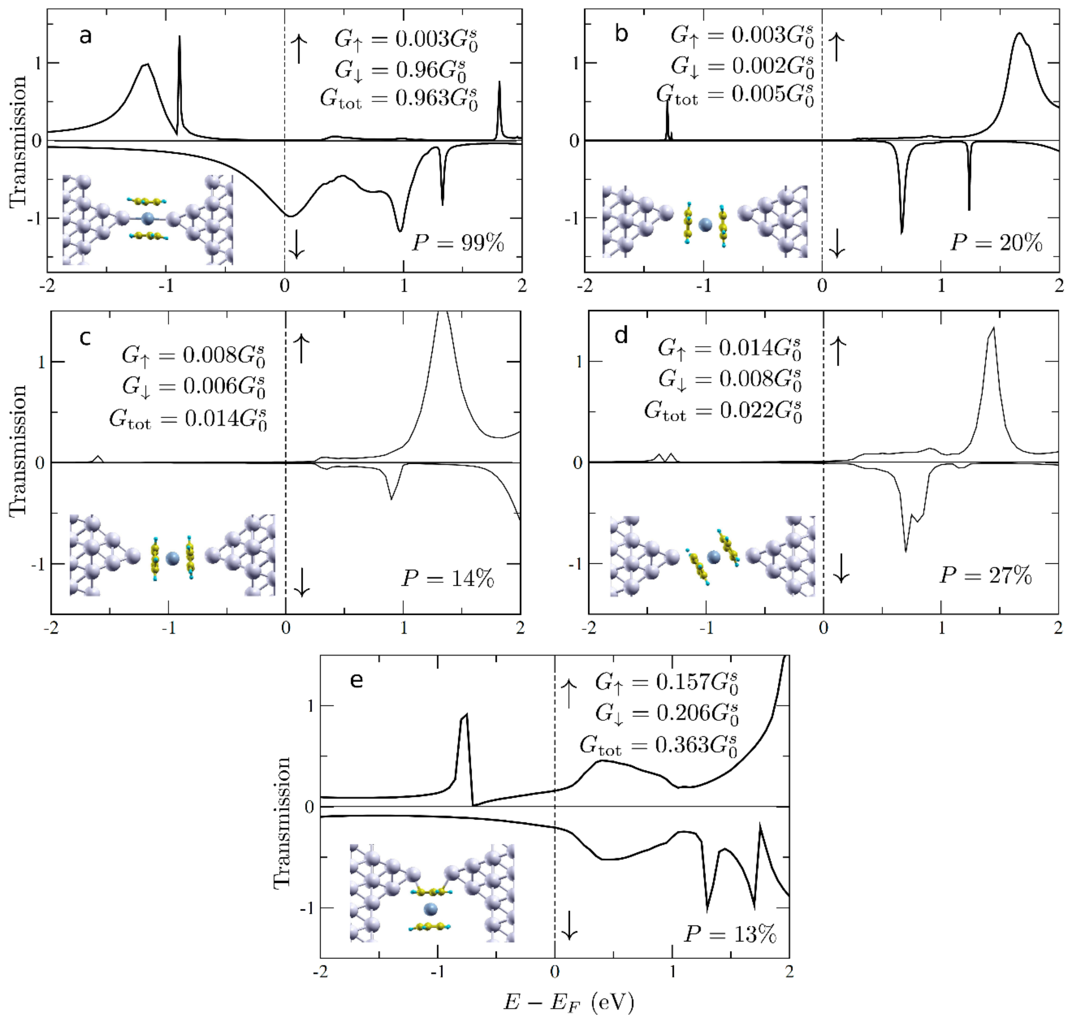

Supplementary Figure 8: Calculated transmission for different molecular configurations (see Insets) in a Ag/vanadocene junction.

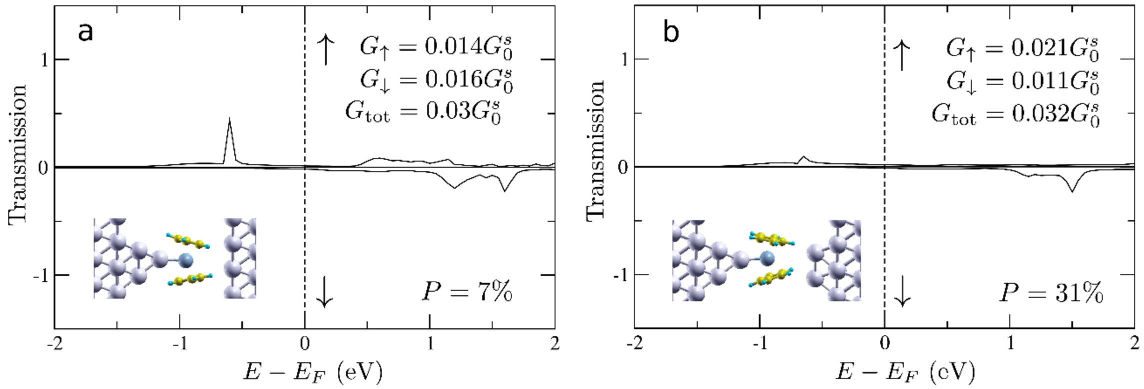

Supplementary Figure 9: Calculated transmission for different electrode structures (see Insets) for a perpendicular Ag/vanadocene junction.

Supplementary Note 8.

Total energy and spin dependent electronic transport of Ag/vanadocene molecular junctions

To verify that the outcome of our calculations is robust and independent of the calculation technique and methodology, we present here additional calculations done using a different methodology with respect to the calculations presented in the main text and in the rest of the Supplementary Information. Specifically, we used the SIESTA[9] and VASP[10] codes for DFT calculations, while the spin-dependent electron transport calculations were performed using a TRANSIESTA code, which is based on non-equilibrium Green's-function (NEGF) formalism with a self-consistent DFT (see Methods).

The relative stability of the parallel and perpendicular Ag/vanadocene junction configurations are presented in Supplementary Fig. 10. While the perpendicular configuration is more stable in general, the parallel configuration is more stable at a larger inter-electrode separation. Note that the most stable perpendicular orientations take place at D=6-6.8 Å, in some deviation from the calculations used in the main text and in Supplementary Fig. 12, in which the minimum energy is obtained at D= 5.8 Å, likely due to different calculation details.

The calculated spin-polarized transmission of a perpendicular Ag/vanadocene junction at different electrode separations is presented in Supplementary Fig. 11. The suppression of spin-up transmission is clearly seen for a wide energy range around the Fermi energy in all cases. In contrast, the spin-down transmission at the Fermi energy is non-monotonic with respect to electrode separation, being maximal (very close to 1) for an intermediate inter-electrode separation of 6.0 Å. This gives rise to essentially perfect conductance spin-polarization, and ideal ballistic spin-polarized transport for this junction geometry.

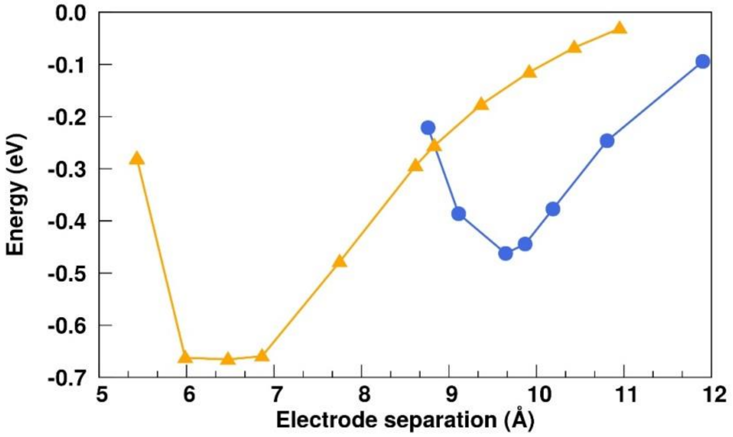

Supplementary Figure 10: Total energy of Ag/vanadocene junctions as a function of electrode separation. The calculations are done for perpendicular (orange triangles) and parallel (blue circles) configurations at different electrode separations. The energy axis was set to zero at the total energy of a particular configuration having two electrodes far away from each other and the molecule at the mid-point, ensuring negligible interaction between the molecule and the electrodes.

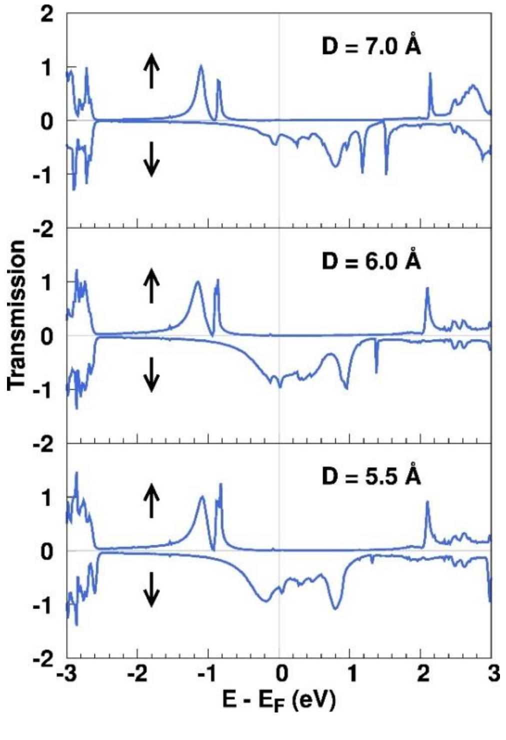

Supplementary Figure 11: Spin-resolved transmission of an Ag/vanadocene junction for perpendicular molecule orientation. Calculated transmission for spin-up and spin-down as a function of energy for perpendicular Ag/vanadocene junction configurations with different inter-electrode separations.

Supplementary Note 9.

Spin-resolved transmissions of perpendicular Ag/vanadocene junctions for different electrode separations

We plot in Supplementary Fig. 12 the spin-polarized transmission for different inter-electrode distance at small separation (D < 6.5 Å). The calculations are done by a homemade tight-binding code[11] with tight-binding parameters extracted from the first principles QE calculations. NEGF formalism was used to compute the spin-polarized transmission (see Methods). Two representative molecular configurations were found, namely a zigzag compact configuration at D < 5.75 Å and a linear configuration at a larger electrode separation (5.75 Å < D <6.4 Å). Note that the two carbon rings are parallel to the transport direction in both configurations. Interestingly, the spin-up transmission is always largely suppressed around the Fermi energy. The spin-down transmission at the Fermi energy, first slightly increases when reducing the inter-electrode separation - from 0.8 at D = 6 Å to almost 1 (i.e., close to ideal ballistic spin transport) at D=5.8 Å, which is the most energetically stable configuration of the junction, discussed in the main text. Then it rapidly decreases to 0.2, when the zigzag configuration is formed, reflecting much weaker hybridization between the vanadocene d_{Z^2} and the s frontier states of the silver electrodes in the last case (D = 5.4 Å).

The calculations presented in Supplementary Fig. 10 and 11, as well as in Supplementary Fig. 12 and the main text were performed using different methods as described in Methods. Still, the main outcome is similar. In particular, the nearly perfect spin-filtering for the most stable perpendicular junction configuration, the significant suppression of spin-up transmission in a very wide energy window around the Fermi energy, and the non-monotonic contribution of spin-down transmission as a function of inter-electrode separation are all clearly reproduced, regardless the calculation method.

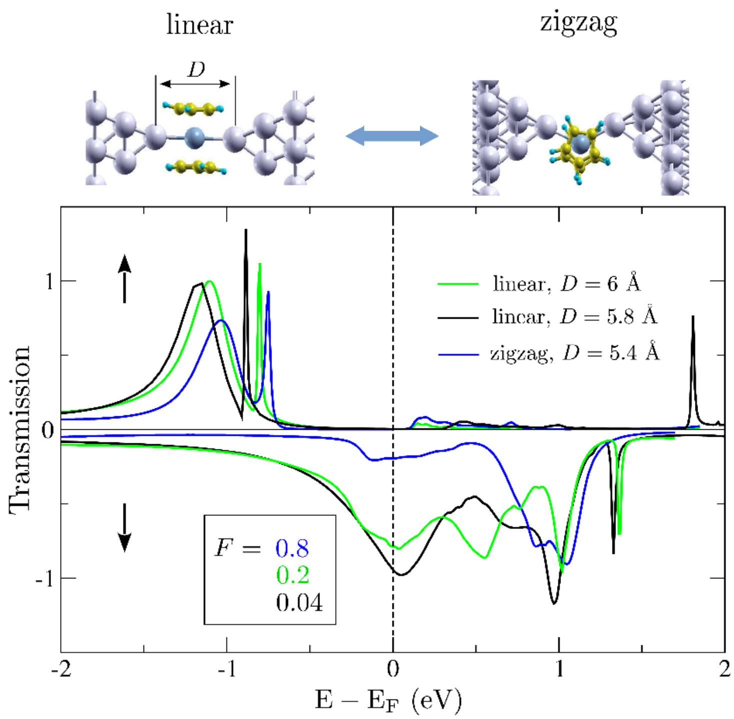

Supplementary Figure 12. Spin-resolved transmission of an Ag/vanadocene junction for perpendicular molecule orientation. Top panel: calculated junction structures for *D* = 6 Å and 5.4 Å (left and right, respectively). Bottom panel: calculated transmission for spin-up and spin-down as a function of energy for three different inter-electrode distances. When the linear junction is squeezed, a zigzag geometry is formed.

Supplementary Note 10.

Interference in terms of vanadium- and carbon-originated molecular states

The relevant interference takes place between the main transport contribution via the vanadium d_{Z^2} level and the transport contributions from many states of the carbon rings located in a wide range of different energies. The latter states are located far from the Fermi energy at different energies, and can be clearly recognized in Supplementary Fig. 13a, at E > 3*eV* and E < -2*eV*. Our analysis (carried by switching off, one by one, different carbon states) has shown that one cannot single out a specific carbon state that dominates the interference. All these states are of importance, contributing to the efficient suppression of transmission at the Fermi energy for spin up, and to the finite phase shift in the even-symmetry scattering channel, as simulated in Supplementary Fig. 16b,c using a simple toy model.

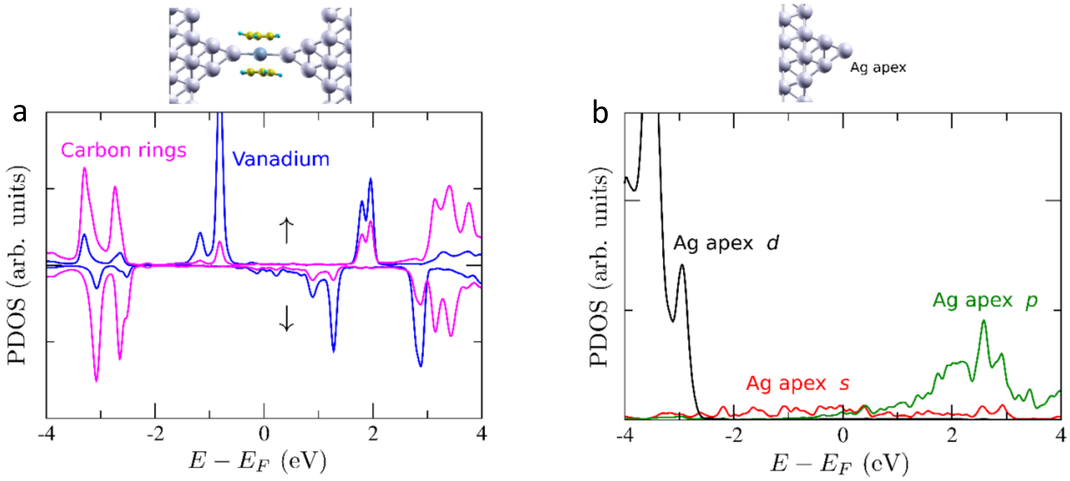

Supplementary Figure 13: Calculated projected density of states. a, Calculated PDOS for Ag/vanadocene junction. The projection is on the π-system of the vanadium's carbon rings (pink) and the dz^2 orbital centered on the vanadium (blue). **b,** Calculated PDOS on different atomic orbitals located on the apex atom of a Ag electrode.

Supplementary Note 11.

The origin of the different widths for spin up and spin down transmission peaks

The different widths of the spin up and down transmission peaks (main text, Fig. 4) can be mainly associated with the different density of states on the apex atom of each silver electrode above and below the Fermi energy, as presented here in Supplementary Fig. 13b. Low s- density of states around E=-1.2 *eV* on the Ag tip apex results in a rather narrow transmission peak for spin up at this energy (main text, Fig. 4c; green curve). In contrast, additional Ag *p*-states appearing at E>0 close to the Fermi energy give rise to a broader transmission feature for spin down electrons at these energies (main text, Fig. 4c; green curve).

Supplementary Note 12.

The origin of the shift in the transmission peaks observed in figure 4e in the main text

In the calculations, when the vanadocene molecule is coupled to the electrodes its levels are renormalized due to the coupling matrix Σ added to the molecule's Hamiltonian H_{mol} as schematically depicted in Supplementary Fig. 14 below. The new eigenvalues of the (non-Hermitian) H'_{mol} are complex. The real term describes renormalized level positions, while the imaginary term (Δ) describes their coupling to the electrodes and defines the level's life time. The coupling matrix *Σ* is defined as *VGV*, where *V* is the hopping matrix between the molecule and the electrodes and *G* is the Green function of (the isolated from the molecule) electrodes. On the right panel of Supplementary Fig. 14 we show again the transmissions presented in the main text in Fig. 4e,

where the positions of the (renormalized) vanadium d_{z^2} - originated level (relevant for transport) are indicated by colored arrows for different coupling strengths. We see that the energy level's position (arrows) is shifted, following very well the position of the corresponding transmission peak. Thus, the shift in the transmission peak reflects change in the coupling strength of the vanadium d_{z^2} orbital to the electrodes for the tree different cases: i) coupling by only vanadium d_{z^2} - orbital to the electrodes (green); ii) coupling to the electrodes by the d_{z^2} - orbital and by the π orbitals of the carbon rings (red); and iii) direct electrode-electrode coupling is added (dashed black). The shift is due to the different coupling matrices Σ: for cases (i) and (ii), simply because of different hopping matrices V, while the electrode's Green function G is given by two independent contributions from left and right electrodes, $G=G_L+G_R$. In case (iii), the last statement is not true due to direct coupling between electrodes, so that $G=G_L+G_R+G_{LR}$ with a nonzero crossing term G_{LR}. To conclude, the shift is related to changes in the hybridization, since in each case (i-iii) different orbital combinations are taken into account, while the asymmetric shape of the transmission peaks is an outcome of the interference discussed in the main text.

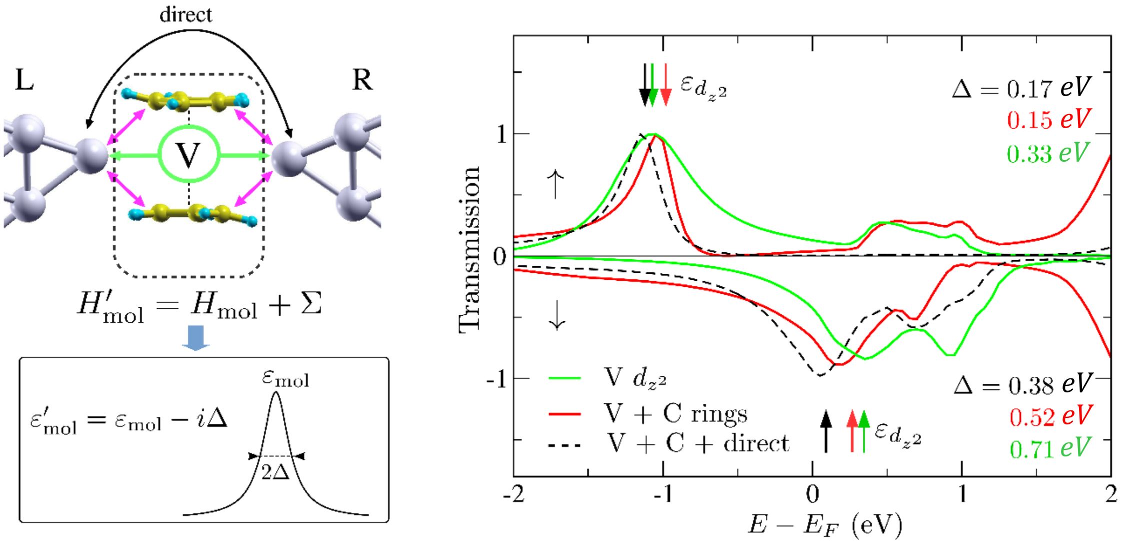

Supplementary Figure 14: The role of hybridization and electrode-molecular levels coupling on the position of the transmission peaks.

Supplementary Note 13.

Toy model for understanding spin-dependent interference

To illustrate the mechanism of spin filtering by quantum interference, we set up a simple tight-binding model shown in Supplementary Fig. 15. It consists of three levels: two levels simulate the energy levels of the carbon rings (at 6 eV) and one spin-split level simulates the spin-polarized *d*-level (d_{z^2}) of vanadium, placed symmetrically (with respect to the Fermi level) at -1 *eV* and 1 *eV* for spin-up and down, respectively. The levels are coupled to two semi-infinite 1D chains, representing the silver electrodes.

When the two pathways are treated separately, no spin filtering at the Fermi level is observed. Specifically, the transmission through the spin degenerate carbon levels (pink lines) is obviously non-polarized, while the transmission through the spin-polarized *d*-level (green lines) gives two spin-split Lorentzian structures with equal transmission at the Fermi energy. In contrast, if interference between the two electronic pathways is taken into account (black lines), a full reflection of spin-up electrons and enhanced spin-down transmission are found at the Fermi energy. Note that in this symmetric case (regarding the *d*-level spin up/down position) the perfect spin filtering at the Fermi energy is purely due to quantum interference since the *d*-level density of states are not spin-polarized.

The actual asymmetric shape of the transmission function due to the above-discussed interference could be readily rationalized in terms of the so-called scattering phase shifts. In Supplementary Fig. 16, we analyze the spin-up case (as spin-down transmission has the same shape). For our single band case, the transmission probability can be expressed[12] as $T=sin^2(\delta_e-\delta_o)$, where $\delta_{e/o}$ are phase shifts in even/odd combinations of two electron waves coming from the left and from the right chains caused by a coupling to the molecule (levels). The reference system (zero phase shifts) is the one in which the two chains are fully disconnected (no molecule). Furthermore, based on Friedel sum rules, $d\delta_{e/o}(E)/dE=\pi\,\Delta\rho_{e/o}(E)$, where $\Delta\rho_{e/o}(E)$ are additional PDOS of even or odd symmetry (with respect to the transport direction), due to the coupling to the molecule.

When only carbon rings are coupled (symmetrically) to the chains, as presented in panels (a-c), a small (even) PDOS on the molecule is generated around the Fermi energy (a) accompanied with slowly increasing in energy δ_e (b). This situation results in a small tunneling transmission around zero energy (c). If the *d*-level is added with symmetric coupling to the left and right chains (as in the present case of a d_z^2 orbital), see panels (d-f), δ_e starts to increase rapidly (e) at the d-level energy, -1 eV, correlating with additional d-level derived PDOS (d); δ_o remains essentially zero. This leads to an increase of transmission to the left of the PDOS peak, while the transmission drops exactly to zero to the right when $\delta_e=\delta_o$ (f). It is interesting to note that if the *d*-level is coupled anti-symmetrically (for example, as in the case of a p_z orbital), (g-i), it would lead to a rapid increase in δ_o (h). Consequentially, the transmission curve would change its shape (I), and drop to zero at the left side of the PDOS peak, remaining finite at its right side.

We can conclude that the nature of interference at zero energy (at the Fermi energy) depends not only on the *d*-level position (with respect to the Fermi energy) but also on its symmetry. Note also that the PDOS on the molecule, on the contrary, is very similar in both cases, (d), (g).

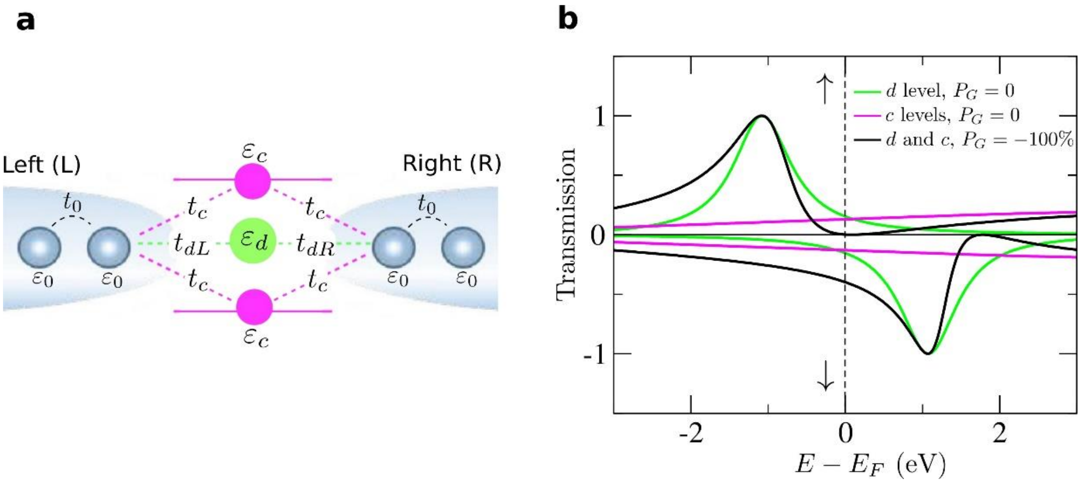

Supplementary Figure 15. Minimal three-level tight-binding model. a, Schematic presentation of the model: *d*-level is set at -1 and at 1 eV for spin-up and down, respectively, c-levels (simulating two carbon rings) are set at 6 eV, hopping integrals are t_{dL} = t_{dR} = 0.8 eV and t_c=1.3 eV while semi-infinite electrodes are described by tight-binding chains, ε_0 = 0 and t_0 = 3 eV. **b**, Spin-dependent transmissions for two conduction pathways: one across the vanadium *d*-level (green) and one across the carbon rings (pink), as well as full transmissions (black), where both pathways are taken into consideration.

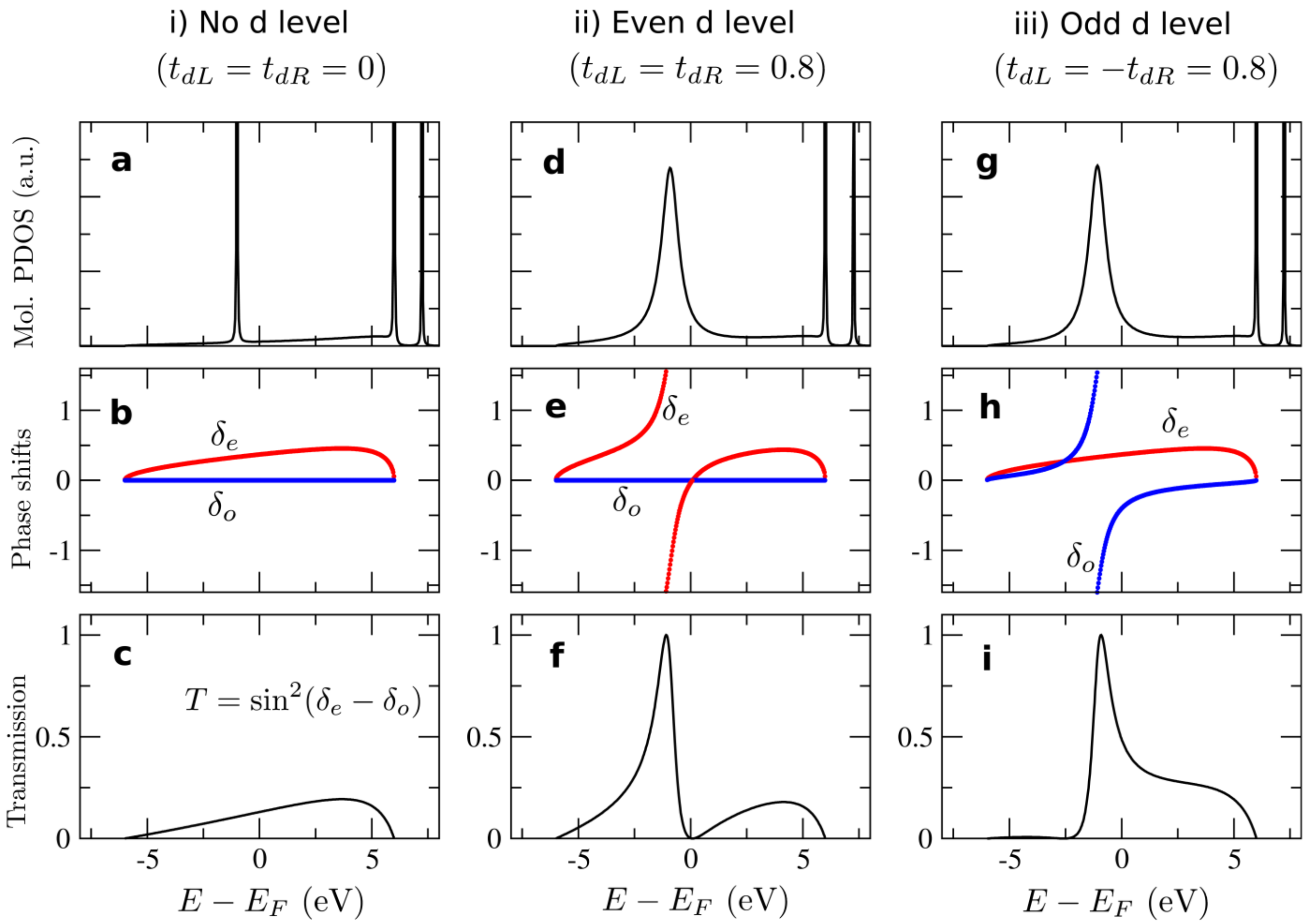

Supplementary Figure 16. Tight binding model for quantum interference patterns in the transmission. Projected density of states on the molecule (sum over all three levels), even/odd phase shifts, and the corresponding transmission functions are shown in the upper, middle, and lower panels, respectively. (i) Only the two c-levels (simulating two carbon rings) are coupled to the semi-infinite chains with t_c = 1.3 eV, (a-c); (ii) A *d*-level of even symmetry is also introduced, where t_{dL} = t_{dR} = 0.8 eV, (d-f); (iii) The "*d* level" has an odd symmetry, where t_{dL} = 0.8 eV, t_{dR} = -0.8 eV, (g-i). The Fermi energy is set to zero.

Supplementary References

1. Blanter, Y. M. & Büttiker, M. Shot noise in mesoscopic conductors. *Phys. Rep.* **336**, 1-66 (2000).

2. Kumar, M., Tal, O., Smit, R. H. M., Smogunov, A., Tosatti, E. & van Ruitenbeek, J. M. Shot noise and magnetism of Pt atomic chains: Accumulation of points at the boundary. *Phys. Rev. B* **88**, 245431 (2013).

3. Roche, P., Ségala, J., Glattli, D., C., Nicholls, J., T., Pepper, M., Graham, A., C., Thomas, K., J., Simmons, M. Y. & Ritchie, D. A. Fano factor reduction on the 0.7 conductance structure of a ballistic one-dimensional wire. *Phys. Rev. Lett*. **93**, 116602 (2004).

4. DiCarlo, L.; Zhang, Y., McClure, D. T., Reilly, D. J., Marcus, C. M., Pfeiffer, L. N. & West, K. W. Shot-noise signatures of 0.7 structure and spin in a quantum point contact. *Phys. Rev. Lett.* **97**, 036810 (2006).

5. Burtzlaff, A., Weismann, A., Brandbyge, M. & Berndt, R. Shot noise as a probe of spin-polarized transport through single atoms. *Phys. Rev. Lett.* **114**, 016602 (2015).

6. Vardimon, R., Klionsky, M. & Tal, O. Indication of complete spin-filtering in atomic-scale nickel oxide. *Nano Lett*. **15**, 3894-3898 (2015).
7. Vardimon, R., Matt, M., Nielaba, P., Cuevas, J. C. & Tal O. Orbital origin of the electrical conduction in ferromagnetic atomic-size contacts: Insights from shot noise measurements and theoretical simulations *Phys. Rev. B* **93**, 085439 (2016).
8. Vardimon, R., Klionsky, M. & Tal, O. Experimental determination of conduction channels in atomic-scale conductors based on shot noise measurements. *Phys. Rev. B* **88**, 161404 (2013).
9. Soler, J. M., Artacho, E., Gale, J. D., García, A., Junquera, J., Ordejón, P. & Sánchez-Portal, D. The SIESTA method for ab initio order-N materials simulation, *J. Phys.: Condens. Matter* **14**, 2745-2779 (2002).
10. Kresse, G. & Furthmüller, J. Efficient iterative schemes for ab initio total-energy calculations using a plane-wave basis set. *Phys. Rev. B*, **54**, 11169 (1996).
11. Autès, G., Barreteau, C., Spanjaard, D. & Desjonquères. M.-C. Electronic transport in iron atomic contacts: From the infinite wire to realistic geometries. *Phys. Rev. B*, **77**, 155437 (2008).
12. Baruselli, P. P., Fabrizio, M., Smogunov, A., Requist, R. & Tosatti, E. Magnetic impurities in nanotubes: from density functional theory to Kondo many-body effects. *Phys. Rev. B* **88**, 245426 (2013).
